# Encapsulating Networks of Droplet Interface Bilayers in a Thermoreversible Organogel

**DOI:** 10.1038/s41598-018-24720-5

**Published:** 2018-04-24

**Authors:** Elio J. Challita, Joseph S. Najem, Rachel Monroe, Donald J. Leo, Eric C. Freeman

**Affiliations:** 1 College of Engineering, University of Georgia, Athens, Georgia 30605 United States; 2 Joint Institute for Biological Sciences, Oak Ridge National Laboratory, Oak Ridge, Tennessee 37830 United States; 3 Department of Mechanical, Aerospace, and Biomedical Engineering, University of Tennessee, Knoxville, Tennessee 37916 United States; 4 College of Engineering, University of Kentucky, Lexington, KY 40506 USA

## Abstract

The development of membrane-based materials that exhibit the range and robustness of autonomic functions found in biological systems remains elusive. Droplet interface bilayers (DIBs) have been proposed as building blocks for such materials, owing to their simplicity, geometry, and capability for replicating cellular phenomena. Similar to how individual cells operate together to perform complex tasks and functions in tissues, networks of functionalized DIBs have been assembled in modular/scalable networks. Here we present the printing of different configurations of picoliter aqueous droplets in a bath of thermoreversible organogel consisting of hexadecane and SEBS triblock copolymers. The droplets are connected by means of lipid bilayers, creating a network of aqueous subcompartments capable of communicating and hosting various types of chemicals and biomolecules. Upon cooling, the encapsulating organogel solidifies to form self-supported liquid-in-gel, tissue-like materials that are robust and durable. To test the biomolecular networks, we functionalized the network with alamethicin peptides and alpha-hemolysin (αHL) channels. Both channels responded to external voltage inputs, indicating the assembly process does not damage the biomolecules. Moreover, we show that the membrane properties may be regulated through the deformation of the surrounding gel.

## Introduction

Membrane mimetics have long been explored for replicating cellular phenomena through the self-assembly of lipid bilayers, including painted bilayers^1^, tethered membranes^2,3^, liposomes^3–5^, bicelles^6,7^, and droplet interface bilayers (DIBs)^8–10^. These systems approximate the functionality of cellular membranes, and provide a platform for studying biophysical phenomena at the cellular level. Understanding the workings of these intricate biological systems offers insights into new approaches for constructing biologically-inspired, autonomic material systems^11^ with applications that range from sensing^12,13^ to actuation^14^ to energy harvesting^15^. DIBS in particular have been proposed as building blocks for such materials^10,11^, owing to their modular properties and simplicity.

**Figure 1:**
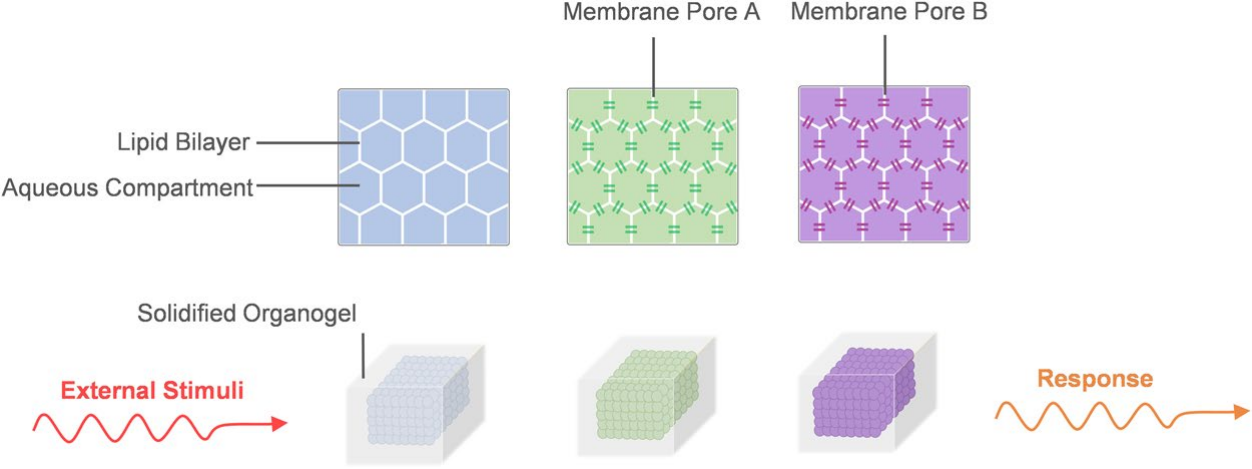
Droplet-based materials. The material consists of networks of aqueous droplets connected by means of insulating lipid bilayers capable of hosting various stimuli-responsive biomolecules. The networks, in their assembly and organization, resemble biological tissues, where each compartment is independent, capable of communicating with neighbouring compartments, and work with other droplets to provide the network with complex functionalities and structural integrity. The material encapsulating the network consists of an oil/SEBS mixture, which is liquid at temperatures higher than 50 °C and solid at room temperature.

The DIB consists of a lipid bilayer formed at the interface of two lipid-encased aqueous droplets in oil^8,10,16^. This is enabled by the amphiphilic nature of the phospholipids, dissolved either in the aqueous phase or the oil phase, which drives the self-assembly of a lipid monolayer at the water-oil interface^10,17^. The resulting lipid membrane represents a model cell membrane capable of hosting various types of transmembrane pores^10^, which could be activated in the presence of a stimulus. Different studies have successfully demonstrated the reconstitution and incorporation of ion channels that can be gated by mechanical^13,18,19^, electrical^20,21^, and optical^20,22,23^ stimuli. Importantly, DIB membranes may be arranged in tailored networks, similar to synthetic tissues^24–26^ (Fig. 1).

Cells in tissues represent independent compartments capable of assembling, communicating, and working together to give tissues their complex functionalities and structural integrity^25–27^. The organization of DIBs in networks approximates these biological tissues, where each droplet may contain different chemicals and biomolecules, communicate, and work with neighbouring droplets to provide the membrane system a wider range of functionalities that could not be achieved using a single DIB interface. The capability of having different chemical constituents and different biomolecules present in each compartment allows for material systems with emergent properties as diverse as in biological tissues. Multiple studies have recently focused on developing rapid and scalable assembly methods^24,25,28^ enabling the construction of tailored and compact structures with increasing functional densities.

However, a key challenge is that unmodified DIB networks are delicate and consequently prone to disruption or degradation outside of a laboratory environment. This limitation was previously addressed by enclosing the droplets in a solid substrate^29^ or in microfluidic chips^30^. Recently, Venkatesan *et al*. addressed this challenge by stabilizing the hexadecane-based oil phase with low concentrations of Poly(styrene-*b*-ethylene-*co*-butylene-*b*-styrene) (SEBS), a thermosensitive copolymer, thus creating soft liquid-in-gel systems for single DIBs^31^. They proved that this method successfully improved the portability and durability of the bilayer system without affecting the bilayer’s properties or its ability to host transmembrane peptides such as alamathecin. Encapsulation of the bilayer membranes has been explored recently with hydrogels as well^32,33^, solidifying the aqueous phases.

Here we present the assembly process of liquid-in-gel, membrane-based materials with synergistic features using the thermoreversible organogels. We combine the mechanical properties of organogels with the versatile functionalities offered by network of bilayer membranes to create a self-supporting, solid material composite with stimuli-responsive capabilities.

We optimize and expand the usage of the SEBS-hexadecane organogel^31^ originally demonstrated by Venkatesan *et al*. to create liquid-in-gel functional materials. SEBS is selected as the support for the DIB networks due to its stability and resilience to degradation compared to other types of polymers^34,35^. The thermosensitivity of the SEBS-hexadecane organogel also provides the benefit of forming lipid bilayers at moderate temperatures while providing a stable solid scaffold at room temperature, allowing for reversible transitions from liquid to gel. We optimized the properties of the organogel for our developed manufacturing process to rapidly create and solidify biomolecular networks.

**Figure 2:**
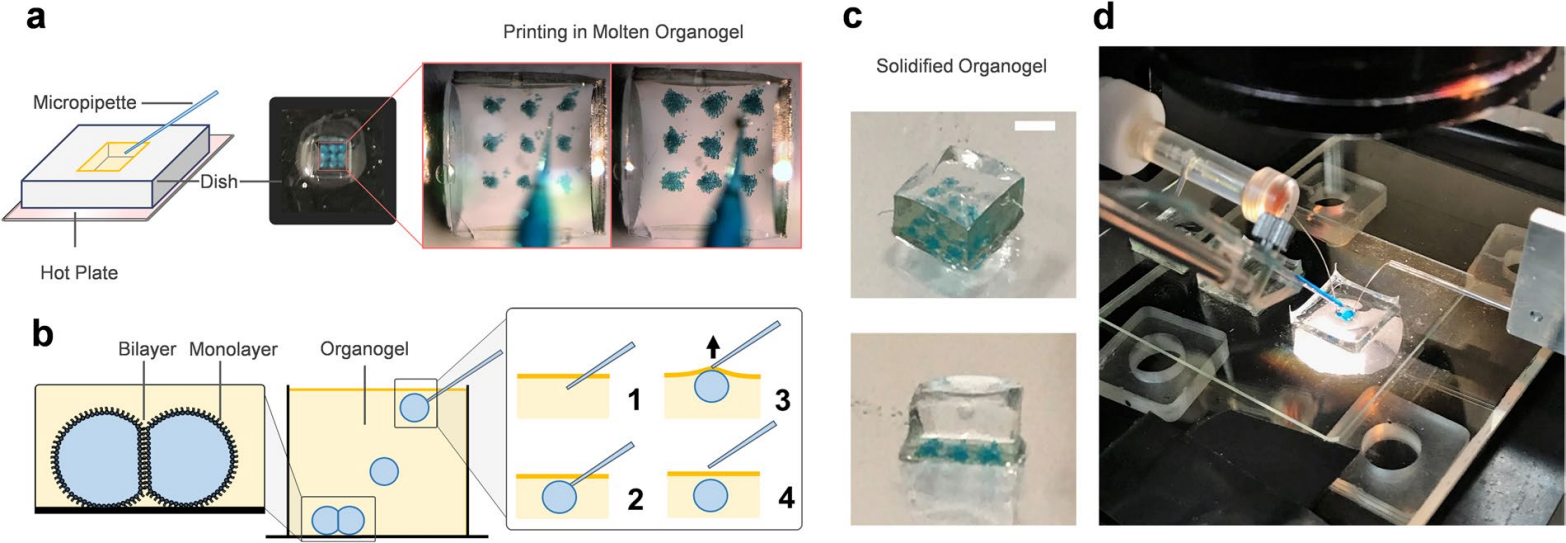
Printing process and resulting liquid-in-gel biomolecular material. (**a**) The printing space consists a polyurethane substrate containing molten organogel and a hot plate to control the printing temperature. We choose polyurethane since it does not absorb oil and it can handle high temperatures. (**b**) Schematics describing the technique used to form and release aqueous droplets. The technique consists of forming a droplet at the tip of the glass capillary and then releasing it by vertically removing the tip from the organogel into air. The process is repeated to generate thousands of aqueous droplets and yields one droplet every 5 seconds (Average ~60 μm in diameter). The glass capillary is mounted on a micromanipulator and connected to a microinjector, which are synchronized and controlled via a single Matlab script. (**c**) The resulting material is removed from the substrate at room temperature. The organogel is solid and maintains the structural integrity of the material. (**d**) Experimental setup during printing. Scale bar: 1 mm.

In this work, mass production of droplets in molten organogel is achieved via a pneumatic-based droplet printing apparatus capable of printing 3D structures of aqueous compartments^36^. The printing apparatus consists of a voltage-controlled pressure clamp connected to a pulled micropipette containing the aqueous solution. The needle is positioned by a 3-axis computer-controlled micromanipulator during printing. A MATLAB script is developed to synchronize the movement of the printing needles with the creation of the droplets. (See Methods, Fig. 2 and Supplementary Fig. S1). Droplets are deposited by lifting the needle out of the oil, separating the droplets by capillary forces. These droplets then fall into place in the molten organogel, form adhesive droplet structures at the bottom of the dish, and are encapsulated in place as the surrounding gel cools.

We produced various encapsulated networks functionalized with different stimuli-responsive biomolecules (Fig. 1). We also demonstrated that the surrounding gel not only can serve as a scaffold to the aqueous droplets but could also act as a solid/physical interface between the liquid networks and their surrounding environment for regulating the membrane dimensions. This provides a modified form of the regulated-attachment method^29^ suitable for larger DIB networks.

## Results

### Design space for fabrication of encapsulated DIB systems and organogel properties

We optimized the viscoelastic properties of the organogel (i.e. SEBS-hexadecane mixture) for repetitive and consistent printing of self-supporting networks with well-defined droplet sizes by adjusting the dissolved SEBS concentration within the hexadecane. Previous studies with SEBS primarily used a 10 mg/mL concentration of the polymer in the oil phase to improve the durability of the DIB networks^31^, but a greater gel stiffness is required for self-supporting structures and for regulating membrane dimensions through compression of the gel. This increase in stiffness is achieved through higher concentrations of SEBS, yet these increases also increase the melt temperature necessary for droplet printing. The elevated printing temperatures may degrade or denature the biomolecules (lipids, proteins, and ion channels), and therefore, a printing temperature less than 60 °C is recommended^31,37–39^. It is essential to find a design space suitable for printing by determining the appropriate range of temperatures and concentrations of SEBS in oil suitable for self-supporting gels, while still protecting the biomolecules from thermal denaturation and retaining DIB functionality during the printing process.

**Figure 3:**
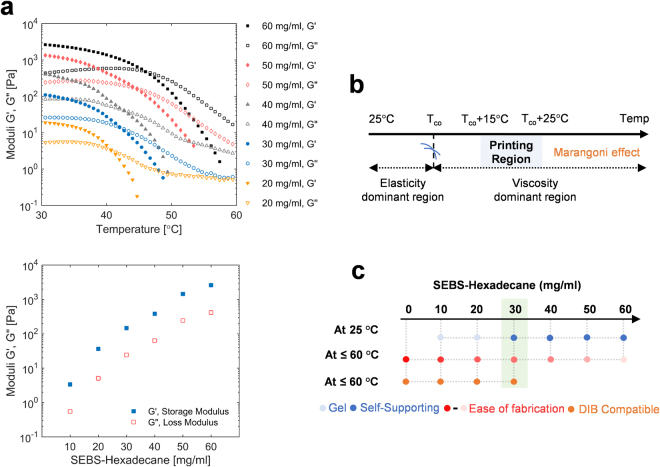
Viscoelastic properties of the organogel at various SEBS concentrations and temperatures. (**a**) A plot showing the change in both the storage and loss moduli of the organogel as a function of temperature for different SEBS concentrations. The results show that at certain temperature, Tco, the modulus of elasticity of the organogel crosses-over from storage to loss. (**b**) The values of both storage and loss moduli increase with the concentrations of SEBS in Hexadecane (25 °C, 1 rad/s). (**c**) Schematic showing the design space parameters taken into consideration when choosing the optimal polymer-to-oil ratio. A concentration of 30 mg/ml was used in this study because it exhibits the most favorable characteristics.

As the SEBS-to-oil ratio increases, the distance between the copolymer micelles decreases and the midblock bridges become less stretched^40–42^. The elastic and viscous moduli increase accordingly by almost an order of magnitude every 10 mg/ml increase in SEBS concentration (Fig. 3a). At a threshold concentration of 30 mg/ml, the organogel forms a soft elastic solid at room temperature (Supplementary Figs S3, S4). It may also be described as a solvent-rich physical structure displaying solid-like characteristics capable of supporting its own weight. At this concentration, the self-supporting solidified organogel has an elastic modulus of around ~120 Pa which is comparable to that of brain tissues and the central nervous system^43,44^. Beyond 30 mg/ml, the polymer-solvent mixture becomes stiffer as both the elastic and viscous components increase (Fig. 3).

An increase in temperature causes the solidified organogel to lose its elasticity which translates into a gradual decrease in its elastic component (Fig. 3). At the cross-over temperature, *T*_*co*,_ the modulus of elasticity, *G’*, of the organogel, becomes equal to the loss modulus, *G”*, and then significantly drops as the temperature increases. At *T* > *T*_*co*_, *G’* of the organogel is now dominated by *G”*, which slightly decreases before asymptotically approaching a plateau. At this point, the organogel is a viscous liquid suitable for creating droplets.

We also observed that at higher temperatures (*T* > *T*_*co*_ + 25 °C) convective flows within the fluid are induced. This results from the creation of a gradient of surface tensions along the liquid surface due to uneven temperature distribution – a thermocapillary phenomenon known as the Marangoni effect^45–47^. Therefore, we found that the ideal temperature range for printing is between *T*_*co*_ + 15 °C and *T*_*co*_ + 25 °C. Operating at this range warrants the creation of complex precise network architectures of microdroplets of consistent sized compartment at suitable temperatures.

With the appropriate concentration of SEBS selected for optimizing stability and printing temperature, we assess the quality of individual membranes with and without the triblock copolymer as shown in Table 1. We measured specific capacitance using the approach developed by Gross *et al*.^48^, pulling the droplets apart and plotting the change in capacitance vs. the change in membrane area and measuring the slope. Values for the specific capacitance at room temperature for hexadecane with and without SEBS were found to be comparable and values at 60 °C show the expected increase in thickness (lower specific capacitance values) associated with additional oil partitioning within the membrane^31,49^, suggesting that the membrane properties are minimally influenced by the presence of SEBS in the oil phase.

**Table 1:** Properties of DIBs formed in varying solvents (n = 5).

Solvent	Specific Capacitance(μF/cm^2^)
Hexadecane (RT)	0.751(±0.06)
Hexadecane (60 °C)	0.534(±0.03)
30 mg.ml^−1^ SEBS-Hexadecane (RT)	0.713(±0.07)
30 mg.ml^−1^ SEBS-Hexadecane (60 °C)	0.609(±0.13)

We have concluded that an organogel with SEBS concentration of around 30 mg/ml^−1^ exhibits favourable characteristics for droplet printing, which makes it a promising candidate for the fabrication of droplet-based materials (Fig. 1). This polymer-to-solvent composition provides an optimal balance between convenient thermoplastic transitions and favourable mechanical properties at room conditions.

For this specific concentration, ideal printing temperature ranges between 55 °C to 60 °C, significantly lower than the boiling point of water and the thermal denaturation temperatures of most biomolecules. It is worth noting that DIB formation might be possible at higher concentrations of SEBS in oil, however, this requires higher printing temperatures (>60 °C).

### Printing DIB networks

**Figure 4:**
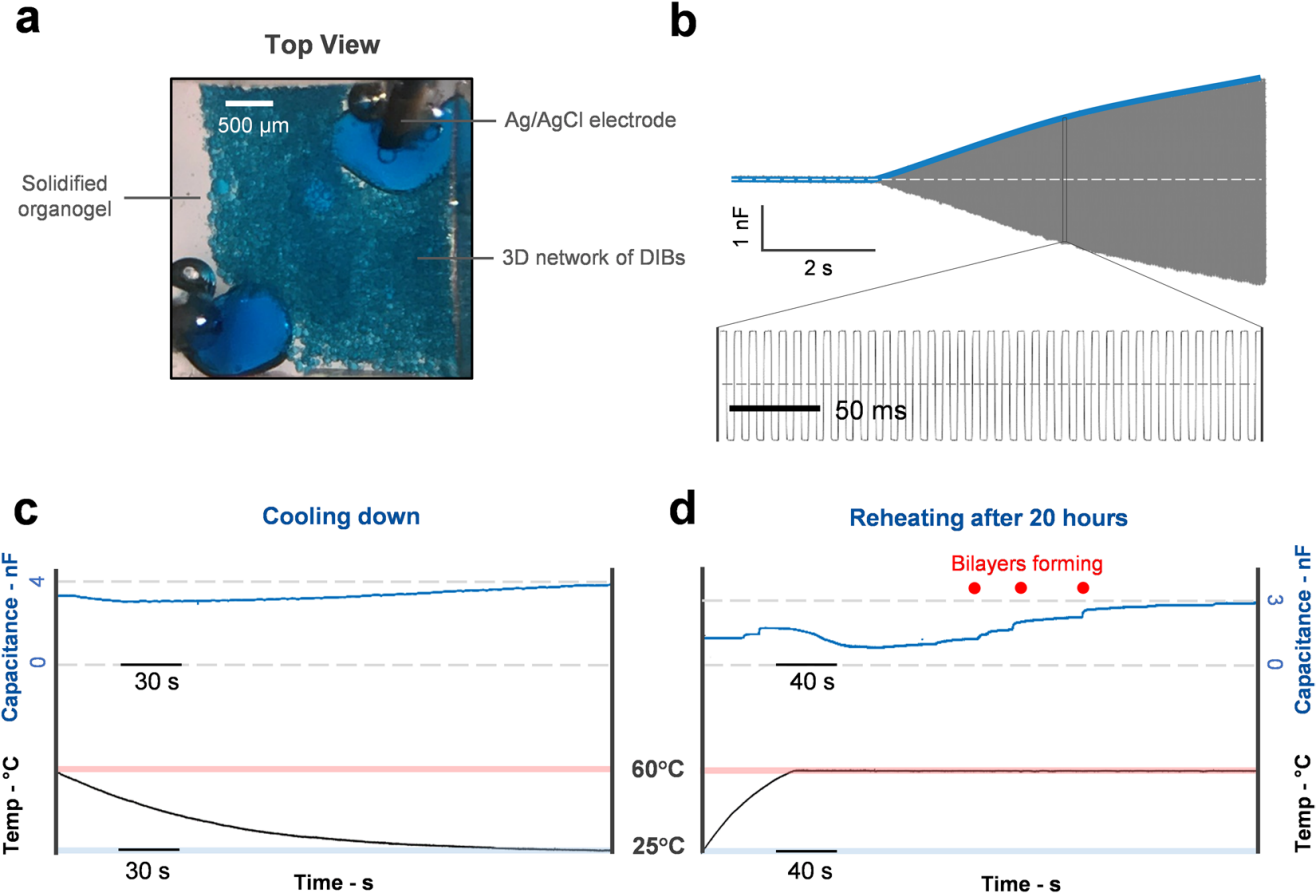
Printing large network of bilayers connected droplets in organogel. (**a**) Top view of the thousands droplets (Average ~100 μm in diameter) connected in molten organogel. The droplets were printed around two larger droplets attached to two Ag/AgCl electrodes. (**b**) The droplets were connected by means of bilayer membranes as demonstrated by electrical capacitance measurements. (**c**) Once the printing is completed, the sample is cooled to solidify the organogel at room temperature. Upon cooling, an increase of 500 pF in the capacitance is observed which could be attributed to the thinning in the bilayers as shown in Table 1. (**d**) 20 hours after solidification, the capacitance of the network decreased almost by half most likely due to the separation of some of the bilayers. Upon heating again, the capacitance increased again due to reformation of some of the bilayers. Scale bar: 500 μm.

Assembling large networks of droplets preserves the fundamental characteristics found in singular lipid bilayer membranes^20,21,50^. From an electrical standpoint, these droplets could be essentially considered as a cluster of resistors-capacitor (RC) circuits linked together in unsystematic configurations (Fig. 4, Supplementary Fig. 2)^10,20,21,24,50,51^. We conducted electrical measurements across a large printed network of encapsulated aqueous droplets (>3000 droplets) using two agarose-coated Ag/AgCl wires and demonstrated the typical capacitive response of bilayer membranes^20,21,50^. Large droplets were formed at the agarose-coated tip of the wires to establish electrical connections with the printed networks similar to previous printing studies^23,24^. When the droplets formed lipid bilayers between the electrodes, we observed a gradual increase in the equivalent capacitance of the network before reaching a steady state value of 3.2 nF (Fig. 4b,c). Upon cooling, the organogel solidifies into a viscoelastic solid-like structure (Figs 2c and 3a), encapsulating the network in their original geometry. In parallel, the amplitude of the capacitive response slightly rises to 4 nF (Fig. 4d). The growth in capacitive current response resulted from the continuous thinning across bilayer membranes (expelling of oil from the hydrophobic core) caused by the overall decline in temperature^52^ as expected from the values in Table 1. The network conductance was negligible for all cases, and minimal membrane leakage was observed.

At room temperature some of the formed bilayers remained stable for at least 20 h after solidification. We demonstrated this by electrical investigation which indicated the persistence of the capacitive current though with a slight decrease in its amplitude to around 1.2 nF. This could either be attributed to select droplet coalescence in the system^53,54^, or reduction in fluid volume through evaporation^55^, both known phenomena in DIB networks. When heated again, the solidified matrix liquefies (Fig. 3a) and allows the coalesced droplet pairs to pursue a new equilibrium. Unconstrained, the deformed aqueous compartments regain their spherical shapes then form new lipid bilayers with adjacent droplets. This is marked by a decrease in capacitance as the gel relaxes, then as by individual increases (Fig. 4) as droplets form new interfaces after coming into contact.

### Incorporation and reconstitution of ion channels

The lipid membrane acts as a near-impermeable seal restraining mass transport among connected aqueous compartments with similar osmotic content^56^. However, these membranes may be functionalized by the inclusion of self-inserting transmembrane pores. A wide variety of these pores and peptides have been incorporated in membrane mimics^57–63^. Here we focus on a pore forming toxin (alpha-hemolysin (αHL)^10,64^) and voltage-gated peptides (alamethicin^11,65^) as these are classically employed in DIB networks and provide points for comparison. These channels enable transport between adjacent droplets when appropriate conditions are met, and allow for stimuli-responsive droplet exchange^20^. Therefore it is crucial that these pores remain functional in the encapsulated networks post-solidification for the creation of synthetic tissues. The activities of these biomolecules are measured at room temperature where the organogel is solidified under anticipated working conditions.

**Figure 5:**
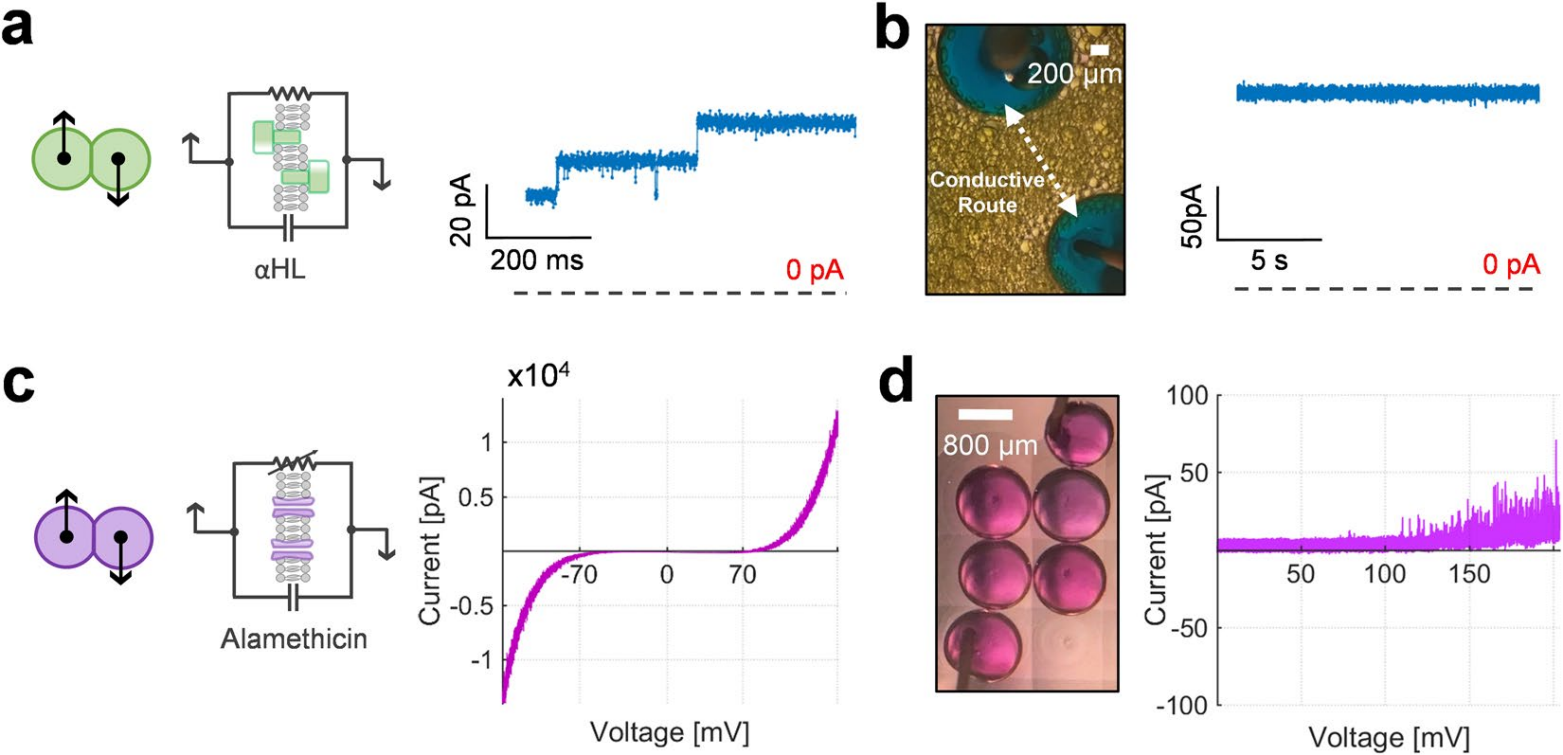
Reconstitution of functional transmembrane pores in encapsulated membranes. (**a**) αHL pores formed at the intersection of bilayer connected droplets allow the transport of ions between the aqueous compartments. The insertion of αHL pores is manifested by a gradual increase in the conductance level across the membranes as a response to a voltage bias. (**b**) Thousands of droplets containing αHL pores (yellow) were printed around the electrodes. These membranes form a conductive route as the ionic current travels across the network, when a constant voltage bias is applied between the electrodes. (**c**) Alamethicin peptides are activated at a voltage threshold around 70 mV and exhibit non-linear current-voltage behavior. (**d**) In a separate experiment, alamethicin peptides were incorporated in a 2-by-2 network configuration. Because the applied voltage bias is divided between the membranes, the activation threshold of alamethicin peptides of the whole network is shifted to higher values. Scale bars: 800 μm.

We begin by introducing αHL into droplets (yellow) forming a DIB. The monomers gradually self-inserted into the bilayer membrane forming conductive pores which enable transport between the adjacent compartments^16,62^. This is electrically manifested by a gradual and discrete increase in the bilayer conductance (Fig. 5a). We found that the conductance (0.38 ± 0.05 nS, n = 5) across a singular bilayer membrane containing αHL (formed in an organogel concentration of 30 mg/mL at 60 °C then brought to room temperature) is in line with results found with membranes formed at room temperature in hexadecane only (0.36 ± 0.06 nS, n = 5)^66,67^. We also confirmed that these αHL conductance levels were unaffected by at least 3 heating and cooling cycles. Therefore, the applied heat coupled with the act of mixing of the aforementioned concentration of polymer with hexadecane did not interfere with the activity of alpha-hemolysin pores in the bilayer membranes at room temperature.

Next, we investigated the activity of αHL in large networks (>2000 droplets). In this case, aqueous inclusions of various sizes containing αHL were printed in the molten organogel around the droplets attached to the electrodes. At an early stage in the network construction (~20 droplets), the insertion and removal of multiple alpha-hemolysin pores inside the network of droplets were recorded at 25 °C similar to the results in Fig. 5a. This corresponds to the discrete variations in the conductance levels when a constant voltage bias is applied across the connected compartments. However, as the network grew larger, the step changes in conductance levels became less pronounced. At this point, subsequent addition of pores are negligible, as the measured current response is across the entire network. This was expected since the change in total conductance per channel insertion rapidly diminishes as the network expands (Supplementary Fig. S2). After printing, the organogel-droplets sample is cooled again to solidify at room temperature. A constant voltage bias applied across the encapsulated αHL imbued network resulted in continuous non-zero current response of around 175 pA with minor fluctuations (Fig. 5b). It is important to note that these encapsulated membranes without αHL remained impermeable, exhibiting minimal conductance similar to the results in Fig. 4.

We repeated the experiments using alamethicin peptides which usually insert into the insulating lipid membrane and aggregate to form a conductive pathway when the transmembrane potential is increased above a certain threshold (~70 mV)^16,62,68^. The behaviour of alamethicin channels was interrogated in single and multiple membranes. Aqueous compartments containing alamethicin (purple) were linked together at high temperature (60 °C) in molten organogel at various configurations systematically forming electrical circuit between the electrodes (Fig. 5c,d). The behaviour of alamethicin channels in solidified bilayers is then explored by cyclic voltammetry (CV) measurements at room temperature. Owing to the voltage-dependent nature of alamethicin, the voltage-current response throughout the network depends on the applied potential across each lipid bilayer. Therefore, the net output depends both on the amplitude of the applied voltage across the bilayer system as well as how it is distributed across the individual membranes.

The CV (n = 3) measurements were taken for a singular membrane containing alamethicin channels using a triangular voltage ramp with a frequency of 10 mHz (Fig. 5c). As the transmembrane potential gradually increases, the likelihood of alamethicin gating increases, yielding a non-linear current-voltage behaviour, similar to a diode or a voltage-dependent resistor^11^. The same pattern was observed when alamethicin channels were incorporated in larger assemblies of droplets. As the applied voltage between the electrodes was divided among the bilayers (Fig. 5d), the probability of alamethicin activation is diminished due to the distribution across the individual membranes^69^. Consequently, alamethicin is less effective for enabling droplet-droplet exchange in larger networks compared to αHL.

The functionality of αHL and alamethicin channels in encapsulated membranes was similar to the ones observed in conventional liquid-in-liquid setups^37,62,70^. These results further confirm that the formed bilayers do not contain any oil or SEBS residues and that the higher printing temperatures did not affect the activity of transmembrane pores^16,62^.

### Mechanical regulation of bilayer membrane: capacitive sensing

Next, we demonstrated that encapsulation in a polymer-based gel matrix allows for mechanical interaction with the environment and regulation of the membrane dimensions. The organogel is employed as a viscoelastic buffer, capable of converting external mechanical perturbations into changes in the membrane dimensions. This is similar to the regulated-attachment method^29^ but with additional flexibility for encapsulated printed networks.

**Figure 6:**
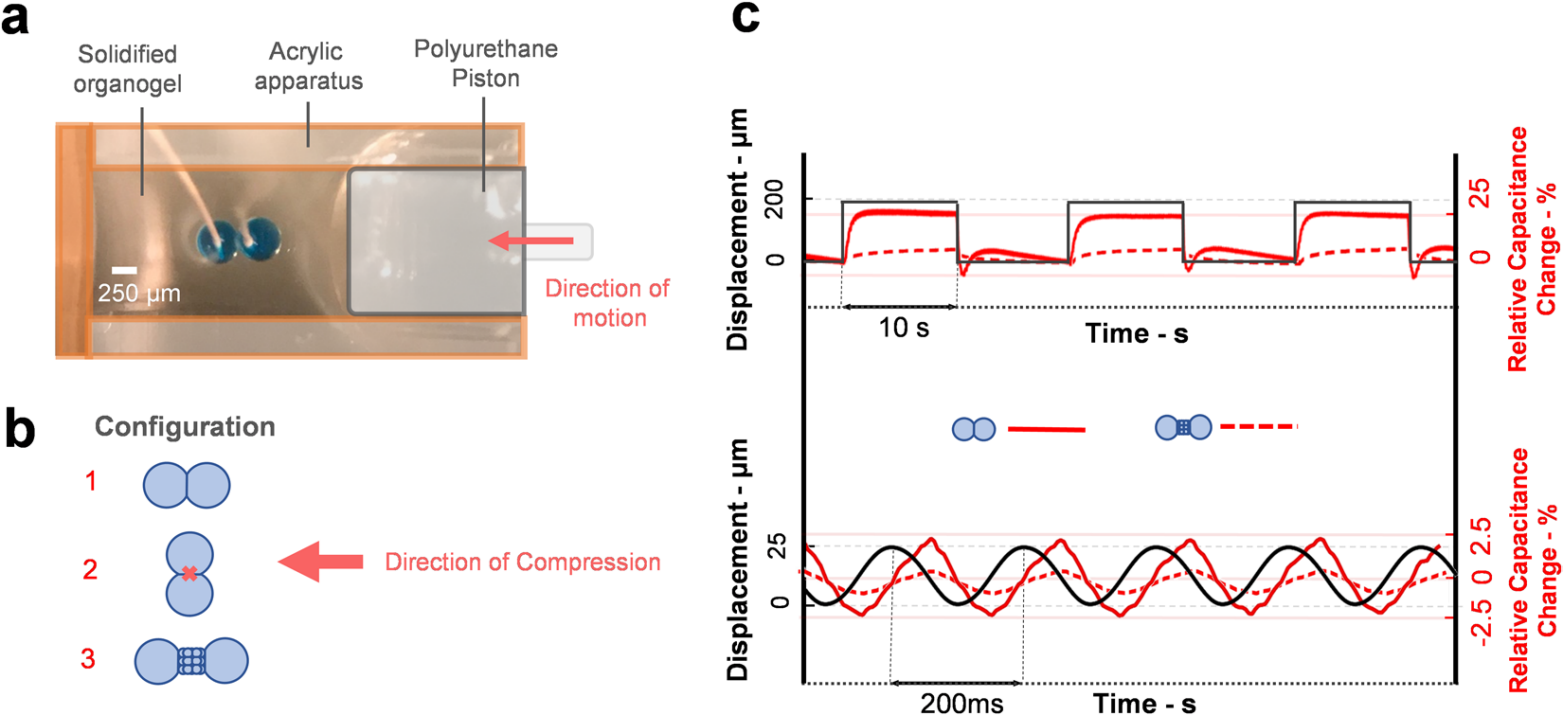
Mechanical functionality of the encapsulated membranes. (**a**) The encapsulation of the lipid membranes adds another functionality to the system. This was tested by systematically applying mechanical perturbation to the solidified organogel using a compression rig. The change in the bilayer was monitored by electrical capacitance measurements. (**b**) We varied the configuration of the droplets with respect to the direction of the mechanical force. In configuration 1 and 2, the mechanical force was perpendicular and parallel to a single membrane respectively. In configuration 3, the mechanical force was applied across a 3D structure of smaller droplets (N = 20 droplets, ~100 μm diameter). (**c**) Applied mechanical vibration showed changes in the bilayers capacitances in configurations 1 and 3, and no changes in configuration 2. Although capacitance variations in config. 3 had the same trend observed in config. 1, the changes were relatively smaller. Scale bar: 250 μm.

In an unconstrained fluid environment, DIB membranes expand or contract until equilibrium is attained^19^. The viscoelastic matrix on the other hand, restrains the interfacial area to the initial dimensions upon gel cooling as demonstrated by electrowetting experiments^31^. Consequently, deformation of the surrounding gel regulates the membrane dimensions. This was investigated by deforming the gel in a cyclical fashion through a piezoelectric actuator while monitoring the capacitance of the DIB network for different configurations of encapsulated lipid bilayers (Fig. 6a).

Three configurations were tested as shown in Fig. 6b. The first two cases examined the dependence on the direction of compression, and the third examined the behaviour of a collection of droplets between the electrodes. Changes in membrane size were observed when the direction was applied perpendicular to the membrane interface (Cases 1 and 3, Fig. 6b). Interestingly, when the compression was applied in the parallel direction (Case 2, Fig. 6b) either minimal change in membrane dimensions or membrane rupture was observed.

A piezoelectric actuator was used to compress the network in a cyclical fashion, both in step displacements (5 mHz, 200 μm) and sinusoidal displacements (5 Hz, 25 μm). The displacement of the actuator was tracked and measured simultaneously with the membrane size as shown in Fig. 6c. Membrane size is recorded as a function of specific capacitance as shown in Supplementary Fig. S5. No changes in membrane permeability were observed during the deformation process.

The step compression produces a 25% increase in the capacitive current as the organogel is compressed by 200 μm in the axial direction. This corresponds to an increase of 20 μm in the membrane diameter. Once the organogel is released, the bilayers area shrinks simultaneously, and overshoots before returning back to its original equilibrium value. The 3D network of droplet exhibited the same response trends when subjected to the various mechanical compression inputs. However, the variations in the capacitive current were less pronounced. The intermediate droplets having relatively smaller diameters (~100 μm compared to ~700 μm for single membrane) renders their subsequent formed interfaces small as well. At the same time, smaller aqueous inclusions are stiffer and harder to deform^71–73^.

The sinusoidal displacement was accompanied by an out-of-phase sinusoidal capacitive response due to the changes in the bilayer size^13,19,74^.This fluctuation in the membrane size had the same frequency of the applied strain and was lagging by approximately $\frac{\pi }{4}$ rad angle. The measured change in the single bilayer capacitance was roughly 2%, with a similarly reduced variation when multiple membranes were involved.

## Discussion and Conclusions

We demonstrate and discuss the encapsulation of large network lipid membranes assembled at the interface of multiple lipid-coated droplets in a thermosensitive organogel. This is an advancement toward autonomic, membrane-based material with emergent properties and improved durability and portability. We optimized the polymer-based organogel to be self-supporting at room temperature while providing adequate conditions for printing aqueous droplets at a higher temperature.

We expanded the functionality of the enclosed network by reconstitution and incorporation of transmembrane proteins and ions channels. The activities of these membrane-based biomolecules, were not affected throughout the assembly process, enabling the creation of networks of membranes with tailored properties. We have also shown that the encapsulation of the droplets in a solid-like medium has other advantages such as regulation of membrane dimensions, enabling the material to act like a capacitive sensor. The result is the successful encapsulation of DIB networks in a self-supporting gel, allowing for regulated-attachment mechanics in DIB networks and improving their durability and portability in non-laboratory settings.

## Methods

### Organogel preparation

The polymer-based organogel is prepared by mixing specific quantities of SEBS (Poly(styrene-b-ethylene-co-butylene-b-styrene, Kraton G-1650E; 10 kg.mol^−1^, used as received) with hexadecane oil at 100 °C and stirring at 500 rpm in a closed vial. Once dissolved, the molten polymer-oil mixture forms a viscous clear liquid. It is then passively cooled down to room temperature (RT, 25–28 °C) at which it solidifies. For printing purposes, the organogel may be reheated back again to its molten state at 100 °C with increased stirring until 500 rpm is reached for up to 25 min then added to the substrate.

### Lipid solution preparation

1, 2- diphytanoyl-sn-glycero-3-phosphocholine phopsholipids (DPhPC) (Avanti Polar Lipids) are suspended in a pH-buffered electrolyte solution (10 mM MOPS, 500 mM KCL, pH = 7) to obtain a concentration of 2 mg.ml^−1^. Unilamellar liposomes are acquired by extruding the resulting lipids-in aqueous solution through a filtering block (pore size 0.1 μm, 10 times, Avanti) followed by sonication (Elma Ultrasonics). The liposome buffer solutions are coloured blue using a standard food dye (Kroger).

### Transmembrane proteins and peptides solutions

Alamethicin peptides from the fungus Trichoderma viridae (A.G. Scientific) are dissolved in ethanol at 5 mg.ml^−1^ and then stored at −20 °C. A solution of 1 μg.ml^−1^ is then obtained by diluting the alamethecin reserved stock in the liposome buffer solution (2 mg.ml^−1^ DPhPC in 10 mM MOPS, 500 mM KCL, pH = 7).

Alternatively, heptameric α-hemolysin (αHL) from Staphylococcus aureus (Sigma Aldrich) are suspended and stored in an aqueous solution at 1 mg.ml^−1^. The αHL stock is then diluted to 1 μg.ml^−1^ with liposome buffer solution (2 mg.ml^−1^ DPhPC in 10 mM MOPS, 500 mM KCL, pH = 7) and stored at 2–8 °C. The alamethicin and αHL solutions are respectively coloured in purple and yellow using standard food dyes (Kroger).

### Rheological Measurements

By applying sinusoidal strain to the sample material, the rheological characteristics of various concentrations of SEBS in oil are probed using a parallel plate rheometer (MCR 302, Anton Paar). The stored elastic energy (storage modulus, *G’*) and the energy lost as heat (loss modulus, *G”*) are measured as a function of temperature and frequency. The temperature dependency of different composition of the SEBS-hexadecane organogels is found by applying a continuous temperature ramp from 25 °C to 80 °C with 0.1 °C steps (10 rad/s angular frequency, 0.5% applied strain). Frequency sweep experiments were also performed by varying the frequency from 0.1 rad/s to 100 rad/s with temperatures changing from 25 °C to 70 °C with 5 °C steps (0.5% applied critical strain).

### Printing in molten organogel

A 3D droplet printer is used to create large networks of aqueous droplets with specific complex architectures within the viscous polymer-solvent matrix at elevated temperatures. It has a pneumatic-driven printing head connected to a pulled glass capillary tube filled with a desired aqueous solution. A High-Speed Pressure Clamp (HSPC, ALA Scientific) is mounted to the printing needle through a silicone tube. The pressure clamp has two functional pumps working in parallel, one of them supplying positive pressure while the other one supplying negative pressure. An NI MyDAQ is used to send a series of voltage pulse to the HSPC, which then translates them into a successive pattern of pressure within the glass capillary during operation. A computer-controlled three-axis motorized manipulator (MCL-3, Lang GmbH Hüttenberg) controls the movement of the printing head. The printing glass capillaries are fabricated in house using a pipette puller (P-1000, Sutter). Custom heat-pull protocols are developed to control the puller thus granting needles with tips with sizes of 5, 10 and 30 microns. The droplet size is controlled by modifying the amplitude and duration of the pressure pulse. The pressure clamp provides pressures spanning from 0 mmHg–200 mmHg, results in a large spectrum of droplet size ranging between 10 to 1000 microns depending on the various parameters of the voltage pulses. Several parameters such as temperature, size of the needle tip and applied pressure factor in determining the resulting droplet dimensions. In this setup, only pressure will be varied to control the droplets dimensions while the other two parameters are going to be fixed. A MATLAB script is developed to synchronize the displacement of the printing needle with the application of the pressure. An ITO slide heater (HI-711Dp, Cell MicroControls) is used to heat the organogel. The heater is regulated by a temperature controller (TC2Bip, Cell MicroControls). A miniature thermistor probe (TH-10 Kmp, Cell MicroControls) is attached to the polyurethane substrate to monitor the temperature of the solution which is recorded via DAQ (Digidata 1550, Molecular Devices).

In this study, we found that needles with tip sizes ranging between 5 to 10 microns in diameter are best to create consistent droplet diameters in the molten organogel ranging from 50 to 150 microns as a function of the pressure magnitude and pulse duration. A calibration step usually proceeds the final printing in molten organogel in a separate dish. During this step, the amplitude and duration of the applied pressure is varied to reach the desired droplet size range for the particular experiment and to ensure consistency.

### Electrophysiology

Ag/AgCl electrodes are prepared by dipping silver (Ag) wires (either 125 μm or 250 μm in diameter, Goodfellow) in bleach for around 30 min. The electrodes are then washed with deionized (DI) water before immersing their tip into a molten solution of 2% agarose (Benchmark Scientific). As it cools, the agarose solidifies thus forming a hydrophilic region at the tip of the electrodes for droplet adherence.

Electrical measurements are taken using a Multiclamp 700B (Molecular Devices) with a Digidata 1550 DAQ (Molecular Devices) at a sampling frequency of 10 kHz and filtered with a 1 kHz low-pass Bessel filter. The Axoscope software (Molecular Devices) is used to record the data which is then analyzed using developed MATLAB scripts.

Specific capacitance was measured by forming a single membrane between two droplets and gradually pulling the droplets apart, measuring the membrane area and finding the slope of the total capacitance vs. membrane area^48^. When measuring the specific capacitance of the organogel at room temperature a single value was calculated as the elastic properties of the organogel restricted droplet separation.

### Mechanical Perturbation

An acrylic-based apparatus was built to test the response of encapsulated droplet networks in organogel to mechanical deformation (Supplementary Fig. S6). The piston-like rig consists of a polyurethane cube (3 × 3 × 3 mm^3^) anchored to the tip of a glass micropipette (O.D. 1 mm). The capillary is connected to a custom fitting device to a flexure-guided piezoelectric actuator (P-601, Physik Instrumente) with a 250 μm range. This actuator is connected to a digital controller (E-709, Physik Instrumente) and function generator (33220A, Agilent) to provide cyclical displacement to the piston.

Two Ag/AgCl electrodes (125 μm in diameter, Goodfellow) dipped in a 2% agarose solution are connected to the electrophysiological equipment (Multiclamp 700B, Molecular Devices). Molten organogel is poured into the chamber and cooled down to room temperature after the aqueous droplets are deposited onto the electrodes. Once solidified, a series of mechanical deformation cycles are applied to the organogel. The displacement of the piston and the membrane size are recorded simultaneously through a DAQ (Digidata 1550, Molecular Devices).

## Electronic supplementary material

Supplementary Information
