# Supplementary material for: Encapsulating Networks of Droplet Interface Bilayers in a Thermoreversible Organogel
Source: Sci Rep. 2018 Apr 24;8:6494. doi: 10.1038/s41598-018-24720-5 (PMC5915452; doi:10.1038/s41598-018-24720-5)
Supplement: Supplementary file 1 — Supplementary Information [file 41598_2018_24720_MOESM1_ESM.docx]

Encapsulating Networks of Droplet Interface Bilayers in a Thermoreversible Organogel

Elio J. Challita^1^, Joseph S. Najem^2,3^, Rachel A. Monroe^4^, Donald J. Leo^1^, and Eric C. Freeman^1^

^1^College of Engineering, University of Georgia, Athens, Georgia 30605, United States, ^2^Joint Institute for Biological Sciences, Oak Ridge National Laboratory, Oak Ridge, Tennessee 37830, United States, ^3^Department of Mechanical, Aerospace, and Biomedical Engineering, University of Tennessee, Knoxville, Tennessee 37916, United States, ^4^College of Engineering, University of Kentucky, Lexington, KY 40506.

**Supplementary Information**

**Supplementary Figure S1: Droplets printing apparatus** **in oil** A glass micropipette filled with the aqueous solution is used to disperse the droplets in molten organogel. It is connected by a silicone tube to a pressure clamp which controlled via NI-myDaQ. The NI-myDaQ is used to send PWM signals that would be interpreted by the pressure clamp and executed into a sequence of positive and negative pressures within the capillary tube. A calibration step proceeds the actual printing, whereby the size of the droplets is measured and regulated in separate polyurethane dish containing a similar organogel solution. A 3-axis manipulator is used to manipulate the micropipette to specific predefined coordinates. A MATLAB script is developed to read an excel sheet containing the coordinates and to synchronize the movement of the manipulator with the creation of droplets^1^. During printing, a hot plate is used to melt the organogel.

**Supplementary Figure S2: αHL activity in large networks of droplets.** Assuming a αHL conductance of ~1nS, the conductance level of a single lipid membrane increases consistently by constant conductance value with the increase in αHL pores in the membranes. However, as the number of droplets containing αHL increase, the subsequent insertion of αHL pores become less pronounced as the number of pores in the network increases. The net conductance is dependent on the overall output of the all the pores combined.

**Supplementary Figure S3: Improved portability by encapsulation using the SEBS-Hexadecane organogel.** At room temperature, the solidified organogel encapsulates the printed droplets and improves their portability. It forms a self-supporting solid allowing better handling of the bilayer membranes, which could otherwise rupture when tilted (given their fluidic nature).

**Supplementary Figure S4: Spectra of 30mg/ml SEBS-Hexadecane at 25°C.** At room temperature, rheological measurements indicate that G' is consistently larger than G” and continuously independent of the oscillation frequency (A typical feature of a gel).^2^

**Supplementary Figure S5: Membrane capacitance during compression.** Current response of a single DIB undergoing mechanical stimulation and a triangle wave voltage. A step displacement with an amplitude of 200 μm is applied to a single DIB. As shown, the bilayer properties are not affected by the mechanical deformation and do not become “leaky” before/during/after mechanical stimulation. (Up to 20 cycles).

**
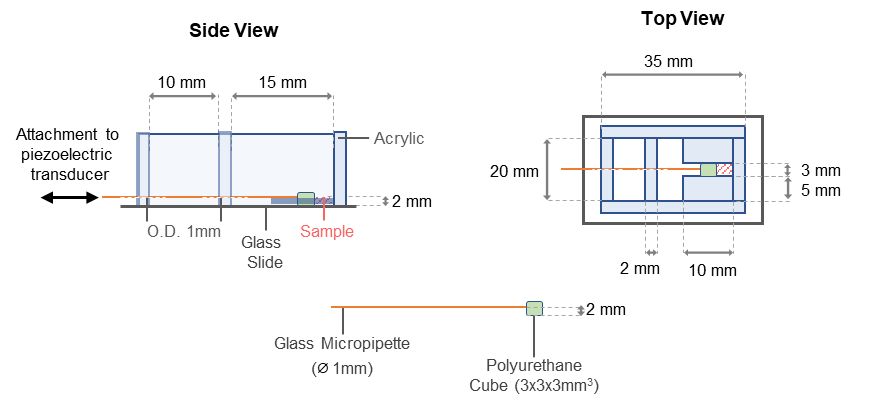
**

**Supplementary Figure S6: Apparatus used for the mechanical vibration experiments.** The illustrated device operates using a piezoelectric transducer to vibrate a glass micropipette rod having a polyurethane cube attached to its top. The micropipette-cube is inserted in a micromachined acrylic-based (PMMA) chamber, and the sample is created in a small groove next to the polyurethane piston. The PMMA chamber is glued to a glass slide. Molten organogel is poured into the sample location and the droplets are printed at high temperature. After printing, the sample is cooled down to room temperature.

**References**

1 J. Challita, E., Najem, J., Freeman, E. & Leo, D. *A 3D printing method for droplet based biomolecular materials*. (2017).

2 Kim, J. K., Paglicawan, M. A. & Balasubramanian, M. Viscoelastic and gelation studies of SEBS thermoplastic elastomer in different hydrocarbon oils. *Macromolecular Research* **14**, 365-372, doi:10.1007/bf03219096 (2006).
